# External Beam Radiation Therapy for Pheochromocytoma and Non-Head and Neck Paraganglioma: A Single-Institution Experience and Systematic Review

**DOI:** 10.3390/cancers18091470

**Published:** 2026-05-02

**Authors:** Katherine S. Jin, Mandy Wan, Kari Chau, Catelina Nguyen, Scott Jackson, Tanaya Kollipara, Evans Whitaker, Erqi L. Pollom

**Affiliations:** 1 Department of Radiation Oncology, Stanford University School of Medicine, Palo Alto, CA 94304, USA; 2 Lane Medical Library, Stanford University School of Medicine, Palo Alto, CA 94305, USA

**Keywords:** paraganglioma, pheochromocytoma, non-head and neck paraganglioma, external beam radiation therapy, stereotactic radiosurgery, stereotactic body radiation therapy

## Abstract

**Background/Objectives**: Paragangliomas (PGLs) and pheochromocytomas (PCCs) are rare neuroendocrine tumors. While external beam radiation therapy (EBRT), particularly stereotactic radiosurgery (SRS) and stereotactic body radiation therapy (SBRT), is established for head and neck PGLs (HNPGLs), its efficacy for non-head and neck PGLs (non-HNPGLs) and PCCs is less defined. We aimed to compare treatment outcomes of EBRT across these anatomic sites. **Methods**: We retrospectively reviewed patients with confirmed PGL or PCC treated with EBRT at a single institution between 1998 and 2025. Treated lesions were classified as non-HNPGL or HNPGL based on the radiation treatment field. Outcomes included local control (LC), distant progression-free survival (dPFS), overall survival (OS), symptomatic/biochemical response, and toxicity. We also conducted a systematic review examining EBRT for non-HNPGLs and PCCs following PRISMA guidelines. **Results**: We included 74 patients with 129 lesions who were treated with EBRT, with 62 HNPGL lesions and 67 non-HNPGL lesions. Of the non-HNPGL lesions, 50.7% (34/67) received SRS/SBRT with a median BED_10_ of 50.8 Gy (range, 35.7–112.5). 5-year LC rate for non-HNPGL lesions was 95.3%, compared to 100% for HNPGL lesions, with 78% of lesions achieving symptomatic control. For non-HNPGL patients, median dPFS and OS were 37.6 and 131 months, respectively. There were no acute or late G ≥ 3 toxicities. Our systematic review (61 studies, 183 patients) showed LC and symptomatic improvement ranges of 76–100% and 76–94%, respectively. **Conclusions**: EBRT, including SRS and SBRT, provides excellent local control and symptomatic relief for both non-HNPGLs and HNPGLs. It represents a safe, effective treatment option for these rare tumors regardless of anatomic location.

## Simple Summary

Due to the rarity of paragangliomas and pheochromocytomas, evidence supporting external beam radiation therapy (EBRT), specifically stereotactic radiosurgery (SRS) and stereotactic body radiation therapy (SBRT), is limited and largely focused on head and neck paragangliomas. This retrospective study examines and compares radiographic, symptomatic, and biochemical responses to EBRT in non-head and neck paragangliomas, including pheochromocytomas. We also conducted a systematic review of EBRT to treat these tumors to contextualize our results within existing literature. Overall, local control was high for both non-head and neck and head and neck paragangliomas treated with SRS/SBRT and conventional EBRT modalities. Our findings validate the safety and efficacy of EBRT including SRS/SBRT, providing a framework for treating these rare tumors regardless of location.

## 1. Introduction

Paragangliomas (PGLs) are neuroendocrine tumors arising from the autonomic nervous system. Sympathetic PGLs originate from chromaffin cells and are often functional tumors, characterized by catecholamine synthesis and secretion. Pheochromocytomas (PCCs) are a subset of sympathetic PGLs located in the adrenal medulla. Conversely, parasympathetic PGLs arise from non-chromaffin cells and are typically non-secretory [1,2,3]. These are rare tumors with a combined estimated incidence in the United States of 500–1600 cases per year [4,5].

Anatomically, these tumors are categorized by location. Head and neck paragangliomas (HNPGLs) are predominantly parasympathetic and most commonly present as carotid body, middle ear, or jugular tumors [6]. In contrast, non-head and neck paragangliomas (non-HNPGLs) are largely sympathetic, often arising within the abdomen, pelvis, and thorax. PCCs are frequently studied alongside non-HNPGLs due to their shared chromaffin cell origin, secretory potential, and location outside the head and neck region [7,8,9,10,11].

While surgical resection has been the gold standard treatment for PGL and PCCs, external beam radiation therapy (EBRT) has emerged as an important alternative or adjuvant treatment, particularly for locally unresectable disease, advanced cases, or to mitigate postoperative morbidity [2,4]. Among EBRT techniques, stereotactic radiosurgery (SRS) and stereotactic body radiation therapy (SBRT) allow for the delivery of highly precise, escalated doses. In a recent meta-analysis including 11,174 patients with 1144 HNPGLs, SRS/SBRT showed excellent local control rates of 94.2% at a median follow-up of 44 months [12]. While emerging data for non-HNPGLs and PCCs are promising, with local control rates between 76% and 100%, evidence remains limited by small sample sizes and a lack of comparative data across anatomic sites [7,8,9,10,11,13,14].

The objective of this study was to evaluate and compare radiographic, symptomatic, and biochemical outcomes of EBRT, including SRS and SBRT, for patients with non-HNPGLs/PCCs versus HNPGLs. Additionally, we conducted a systematic review of EBRT for non-HNPGLs/PCCs to consolidate the existing literature and provide a broader clinical context for our institutional results.

## 2. Materials and Methods

### 2.1. Study Participants and Inclusion Criteria

We retrospectively reviewed clinical charts of patients with confirmed PGL and PCC who received EBRT at our institution from 1998 to 2025. PGL lesions were defined as discrete tumor targets identified on imaging and clinical documentation. Each lesion, primary or metastatic, treated at our institution was included as a separate lesion-level observation. Three non-HNPGL patients and one HNPGL patient had lesions irradiated at outside institutions prior to radiation at our institution. For the analysis, we excluded lesions done at outside institutions due to lack of follow-up details, while subsequent EBRT treatment courses done at our institution were included. We excluded any patients with no follow-up after treatment. This study was conducted with the Institutional Review Board’s approval.

### 2.2. Classification of HNPGLs, Non-HNPGLs, PCCs, and SRS/SBRT

Lesions were classified as HNPGL and non-HNPGL based on the anatomic site of radiation treatment, regardless of primary tumor origin. HNPGLs included carotid body tumors, middle ear PGLs, jugular tumors, as well as other less common sites of HNPGLs such as the vagus nerve, nasopharynx, nasal cavities, paranasal sinuses, larynx, thyroid gland and orbit [6]. Non-HNPGLs included all other locations. PCCs, due to their location in the adrenal medulla, were also categorized as non-HNPGLs.

Patient-level categorization was also based on the anatomic site of first EBRT treatment. Two patients received EBRT to both HN and non-HNPGL lesions at different timepoints and were categorized as HNPGLs based on their first treated site.

EBRT treatments were categorized as SRS/SBRT and non-SRS/SBRT based on treatment technique and regimen. Techniques included, but were not limited to, CyberKnife, Stereotactic Ablative Body Radiotherapy, Volumetric Modulated Arc Therapy, Intensity-modulated radiation therapy, and conventional EBRT.

### 2.3. Data Collection

Patient charts were reviewed for lesion-specific and treatment-related details. Dose selection was determined by the treating physician, based on tumor size and location and guided by normal tissue constraints. Genetic profiles were assessed via next-generation sequencing, clinical documentation, and family history. In patients with elevated baseline urinary fractionated metanephrine (UMN), plasma free metanephrine (PMN), and chromogranin A (CgA), post-treatment catecholamine levels and biochemical responses were tracked, with values reflecting the first follow-up measurement after EBRT. To further evaluate treatment efficacy and safety, data were collected on subsequent therapies, symptomatic response, local and distant progression, radiation toxicities, follow-up duration, and overall survival.

### 2.4. Treatment Details and Response

Local and distant control were defined by the absence of radiographic progression based on imaging reports and physician review at the time of last follow-up. Median follow-up, local progression, distant progression-free survival, and overall survival were calculated in months starting from the final date of EBRT. For median follow-up, the endpoint was last clinical contact or death; for local and distant progression, it was the first imaging confirming progression; and for overall survival, it was the last known contact or death.

Symptomatic response was assessed by comparing baseline clinical notes to symptoms at the last follow-up, with lesions categorized as improved, stable, or worsening. Lesions were classified as “improved” if at least one primary baseline symptom improved or resolved while other symptoms remained stable, and no new radiation-related (acute or late grade ≥ 3) toxicity was reported. Lesions were “stable” if there was no change in baseline symptoms. Lesions were classified as “worsening” if any primary baseline symptom increased in severity or the patient developed a new radiation-related (acute or late grade ≥ 3) toxicity. Symptomatic control was defined as the combination of improved and stable lesions. Radiation-related toxicity was graded using the Common Terminology Criteria for Adverse Events (CTCAE) at acute (within 3 months post-EBRT) and late (after 3 months) timepoints.

### 2.5. Statistical Analysis

Statistical analyses were performed in R, version 4.5.2. Lesion and patient characteristics were calculated as *n* (%) or median [range] for non-HNPGLs and HNPGLs but were not compared with statistical tests. Local progression was estimated using Fine–Gray cumulative incidence curves, with death treated as a competing risk. Distant progression-free survival and overall survival were estimated using the Kaplan–Meier method. Estimates and confidence intervals were calculated at various time points, and the number at risk is also presented. 

Differences between survival distributions were evaluated using the log-rank test and the Fine–Gray test for cumulative incidence functions. All statistical tests were considered statistically significant at *p*-value < 0.05.

### 2.6. Systematic Review of Literature

This systematic review conforms to the Preferred Reporting Items for Systematic Reviews and Meta-Analyses (PRISMA) 2020 guidelines [15]. The study protocol was prospectively registered in the International Prospective Register of Systematic Reviews (CRD420251134126). On 25 September 2025, two authors (K.S.J., E.W.) performed a comprehensive search of PubMed/MEDLINE, Scopus, Embase, and Web of Science. Search strategies were optimized for each database using MeSH terms and keywords including “pheochromocytoma,” “paraganglioma”, “external beam,” “radiotherapy,” “stereotactic radiotherapy,” “CyberKnife,” “proton beam therapy,” “3D conformal,” “intensity- modulated,” “Gamma Knife,” “linear accelerator,” combined with Boolean operators (“AND” and “OR”). One example of a search strategy used is in Appendix A, and full search strategies for each database are available in our PROSPERO protocol. Additional eligible studies were identified through manual citation mining.

Two authors (K.S.J., M.W.) performed the screening and data extraction, resolving any discrepancies through consensus. Inclusion criteria required: (1) non-HNPGLs or PCCs treated with EBRT; (2) full-text availability in English; (3) reporting of tumor-specific outcomes such as local and symptomatic response. Exclusion criteria were: (1) studies where treatment and outcomes for non-HNPGL or PCC lesions could not be disaggregated from HNPGL data; (2) systematic reviews, meta-analyses, editorials, clinical trial registries, or conference abstracts; (3) non-human participants. For all eligible articles, we extracted demographic variables, genetic mutations, tumor characteristics, and EBRT parameters. Primary clinical endpoints included local and distant control, symptomatic and biochemical response, and radiation-related toxicities.

## 3. Results

### 3.1. Patient, Lesion, and Treatment Details

Patient and lesion characteristics are summarized in Table 1 and Table 2, respectively. We included 74 patients with 129 lesions treated with EBRT, comprising 62 HNPGL lesions and 67 non-HNPGL lesions (16 of which were PCC lesions). The median age of patients at diagnosis was 52.2 years (IQR, 17.8–82.0). Among non-HNPGL patients, 35.3% of patients had SDHB mutations and three patients had a family history of PGL/PCC.

The majority of non-HNPGL lesions were of the bone (*n* = 57, 85.1%). The other ten sites included the mediastinum, retroperitoneum, lymph nodes, and lungs. For HNPGL lesions, 82.3% (*n* = 51) were of the jugular foramen, while the remaining 11 sites were of the carotid body, vagus nerve, tympanic cavity/middle ear, and unspecified neck. Notably, 88.1% (59/67) of non-HNPGL lesions were metastatic, compared to only 1.6% (1/62) of HN lesions.

The median biologically effective dose (α/β = 10) (BED_10_) of all treatments was 43.2 Gy (range, 14.4–112.5). For non-HNPGL lesions, the median BED_10_ was 45.9 Gy (range, 14.4–112.5) while HNPGL lesions received a median BED_10_ of 43.2 Gy (range, 18.9–70.4). SRS/SBRT was used for 50.7% (34/67) of non-HNPGL lesions and 91.9% (57/62) of HNPGL lesions. The use of SRS/SBRT increased over time for non-HNPGLs, with only 3% of lesions treated with SRS/SBRT in 1998–2005, but 25.4% in 2016–2025. Among the non-HNPGL lesions treated with SRS/SBRT, median BED_10_ was 50.8 Gy (range, 35.7–112.5). The most frequent regimens were 16–25 Gy in 1 fraction (55.9%) and 21–45 Gy in 3 fractions (23.5%). For the non-SRS/SBRT treatments, median BED_10_ was 39.0 Gy (range, 14.4–72.0), most commonly 30 Gy over 10 fractions (45.5%).

### 3.2. Treatment Response: Biochemical and Symptomatic

Among the symptomatic non-HNPGL lesions with symptom follow-up, 78% (39/50) had improved or stable symptoms, compared to 95.6% (43/45) of symptomatic HNPGL lesions.

Regarding biochemical response (Table 3), four of five patients with elevated baseline CgA showed post-treatment improvement. Both patients with elevated baseline PMN levels demonstrated a decrease following EBRT. No patients presented with elevated UMN levels.

### 3.3. Treatment Response: Progression and Survival Outcomes

Median follow-up was 75.7 months (range, 3.4–275.4) for the total cohort, 36.4 months (range, 3.4–132.3) for non-HNPGL patients, and 88.3 months (range, 4.6–275.4) for HNPGL patients.

Within the non-HNPGL subgroup, 5-year LC rates were similar between SBRT (95.6%, 95% CI, 81.0–99.7) and non-SRS/SBRT modalities (94.9%, 95% CI, 78.5–99.7; *p* > 0.9). Local progression occurred in only four lesions, all of which were non-HNPGLs: two primary tumors (one adrenal PCC, one mediastinal PGL) and two spinal metastases. Both primary lesions achieved LC following salvage radiation. The median time to local progression was 46.7 months (range, 31.8–115.5). There was no significant difference in median BED_10_ between lesions with LC versus those with local progression (*p* = 0.60).

**Figure 1:**
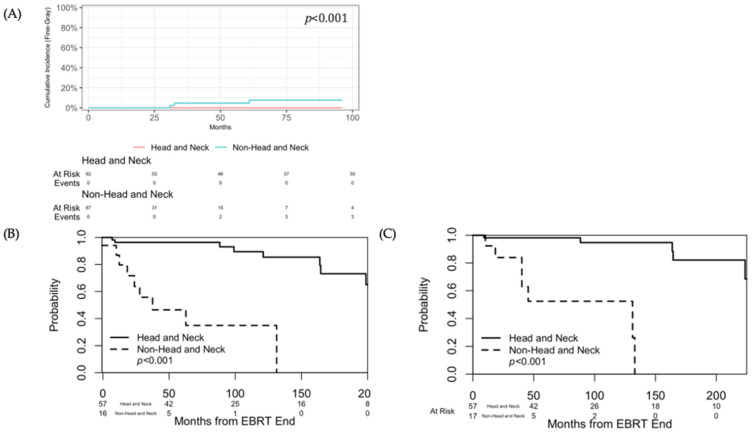
(**A**) Cumulative incidence of local progression. (**B**) Distant progression-free survival. (**C**) Overall survival. Abbreviations: EBRT, external beam radiation therapy.

Both the 5-year dPFS and OS between non-HNPGL and HNPGL patients were significantly better for HNPGL patients (*p* < 0.001) (Table 4, Figure 1).

### 3.4. Toxicity

No acute or late grade 3 or higher radiation-related toxicities were observed. One patient, who had four spinal vertebral metastases treated with SRS, experienced acute G3 dysphagia that persisted and worsened. Clinical notes reported this to be related to overall worsening disease progression of primary HNPGL rather than radiation-induced. The four lesions were all locally controlled.

### 3.5. Systematic Review

**Figure 2:**
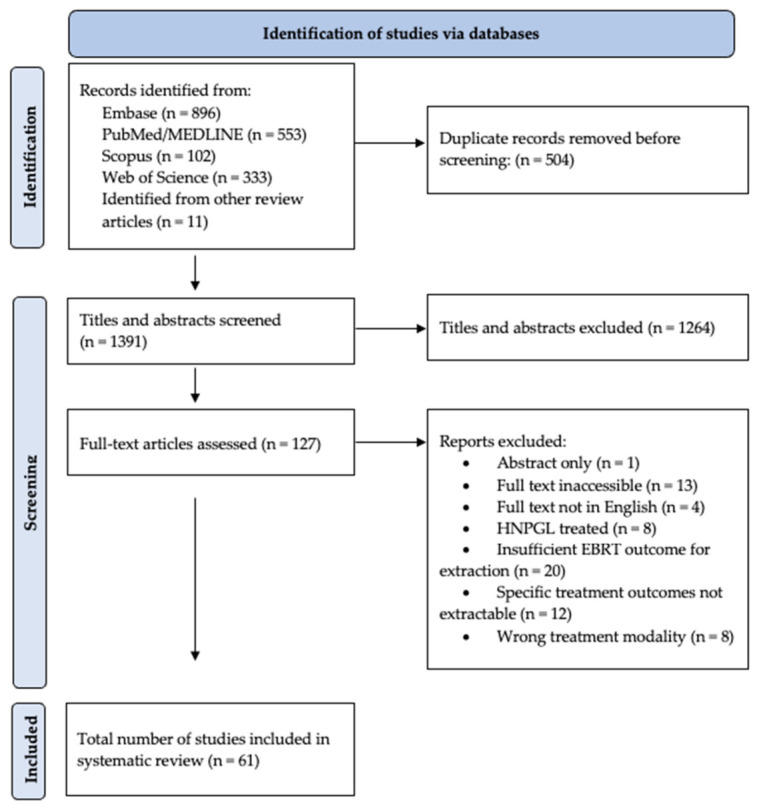
PRISMA flowchart. Abbreviations: HNPGL, head and neck paraganglioma; EBRT, external beam radiation therapy.

Our initial search yielded a total of 1885 articles, including 11 additional studies identified via citation mining. After removing duplicates and screening titles/abstracts, 127 full-text articles were reviewed, and 61 met inclusion criteria (Figure 2). This included 183 patients (334 lesions): 73 patients from 54 case reports and 110 patients from seven cohort studies.

Case reports are summarized in Table 5. Osseous metastases accounted for approximately 70.9% of lesions. The most common treatment course was 30 Gy in 10 fractions, followed by 50 Gy in 25 fractions. LC was achieved in 78.2% of reported lesions at a median follow-up of 24 months. For the 10 lesions treated with SRS/SBRT that reported LC, 60% achieved LC at a median follow up of 23.5 months. Approximately 82.4% of the case reports described symptom improvement. Distant progression was seen in 30.1% of patients, and 75% of patients with biochemical data showed improvement or normalization.

Cohort studies are reported in Table 6. Median ages ranged from 33 to 58 years, and the most treated lesion was the bone (70.1%). LC ranged from 76% to 100% at a median follow-up range of 11 to 53 months. SRS/SBRT was used in three studies, reporting a LC of 94% to 100%. Symptom improvement ranged from 76% to 94%, and biochemical improvement was seen in all the reported series. 1- and 5-year OS ranged from 71% to 100% and 25 to 65%, respectively. Two studies reported Grade 3 toxicities including upper gastrointestinal/esophageal-related toxicities and neuropathy of sciatic nerves. No acute or late grade 4 or higher toxicities were reported.

## 4. Discussion

This retrospective analysis provides a rare, direct comparison of EBRT outcomes between HNPGLs and non-HNPGL/PCCs. While HNPGLs have historically dominated radiation research, our data highlights the efficacy of SRS/SBRT in managing non-HNPGL lesions—a cohort where evidence is traditionally sparse. Across both groups, EBRT achieved excellent local control (LC), with no documented acute or late treatment-related toxicities.

As expected, most HNPGLs were treated with SRS/SBRT (91.9%), demonstrating 100% LC and 95.6% symptomatic control. No HNPGL lesions experienced local failure over a median follow-up of seven years, reinforcing existing literature that establishes SRS/SBRT as a durable treatment standard for these tumors [12].

Our systematic review contextualizes these results. While case reports were heterogeneous, they consistently identified osseous metastases—particularly of the vertebral spine—as the most common non-HNPGL treatment sites, a finding mirrored in our cohort (85.1% bone) and other cohort studies (70.1%). Our symptomatic control rate (78%) sits at the lower end of the range reported in the literature (76–94%), which likely reflects variations in follow-up duration and the high metastatic burden in our patient population.

Our 5-year LC rate of 95.3% for non-HNPGLs treated with SRS/SBRT was similar to smaller series by Vogel et al. and Breen et al., which reported 100% LC for SRS/SBRT-treated lesions [8,9]. Prior studies have reported that higher doses of EBRT are associated with improved outcomes. For example, Breen et al. reported that lesions treated with a median BED_10_ ≥53 Gy had a significantly higher 5-year LC rate of 91% compared to lesions treated with a median BED_10_ < 53 Gy at 62%. Gu et al. reported that 100% of patients with advanced primary tumors experienced LC at a higher dose of 74.4 Gy [10]. In our cohort, SRS/SBRT delivered a significantly higher dose than conventional EBRT (median BED_10_ 50.8 Gy versus BED_10_ 39 Gy). While the higher LC and symptomatic control rates in the SRS/SBRT group did not reach statistical significance—likely due to limited sample size—these trends emphasize the potential benefit of dose escalation tailored to tumor size and metastatic site.

Despite similar LC rates, patient-level outcomes diverged significantly between groups. The 5-year distant progression-free survival and overall survival for non-HNPGL patients (46.5% and 52.5%) were markedly lower than those for HNPGL patients (96.3% and 98.2%). Our 5-year OS rate falls within the range reported by the only two prior studies to have reported 5-year OS (25–65%), both of which exclusively examined metastatic lesions [9,13]. This discrepancy between non-HNPGLs and HNPGLs reflects the aggressive systemic nature of non-HNPGLs and PCCs, which have a metastatic potential of 10–40% compared to the largely localized behavior of HNPGLs [70,71,72]. Indeed, 88.1% of our non-HNPGL lesions were metastatic, and most patients who experienced distant progression already had disseminated disease at the time of their first EBRT. Thus, mortality in the non-HNPGL group appears driven by systemic disease progression rather than EBRT failure.

Given the poor prognosis of metastatic PGL/PCC, management requires a multimodality approach including surgery, chemotherapy, targeted radionuclide therapy, and palliative radiation [4]. Most of our non-HNPGL patients received systemic therapy, underscoring the role of EBRT as a localized adjunct within a comprehensive systemic strategy. Furthermore, the high prevalence of *SDHB* mutations in our non-HNPGL cohort (all of whom had metastatic disease) reinforces the necessity of genetic testing to identify patients at higher risk for recurrence and malignancy [73].

Our biochemical outcomes were restricted by the limited number of patients who had biochemical testing prior to and following EBRT. Despite this, we found that all five non-HNPGL patients (two of whom were PCC patients) had at least one elevated baseline biochemical marker (CgA and PMN). Four of five patients demonstrated a positive response following EBRT, suggesting a potential beneficial impact of radiation therapy on catecholamine secretion. Given excess catecholamine secretion can contribute to hypertension, perioperative hemodynamic instability, and cardiomyopathy, biochemical response to EBRT may have clinical significance beyond LC [47,74,75,76].

Our study has several limitations. The retrospective nature of this study inherently limits the reliability of symptom and toxicity data due to risks of under-reporting or inconsistent documentation in historical clinical notes. Second, our cohort included patients treated over a span of 27 years, which introduces heterogeneity in treatment regimens over time. This is a limitation to our systematic review as well, with included studies spanning over the course of 47 years. Third, due to the small number of non-metastatic non-HNPGL lesions, we did not compare lesion- and patient-level outcomes between metastatic versus non-metastatic lesions. Future studies that examine the role of EBRT between metastatic and non-metastatic lesions would help clarify the role of EBRT and systemic disease progression on overall survival. Furthermore, the lack of standardized, longitudinal post-EBRT biochemical monitoring remains a limitation; future prospective studies are needed to more precisely characterize the impact of radiation on catecholamine secretion. Despite these constraints, our findings provide a comprehensive analysis of non-HNPGL and PCC characteristics, offering a critical foundation for investigating the expanding role of SRS and SBRT in the management of these rare tumors.

## 5. Conclusions

EBRT provides durable local control and symptomatic relief for both HNPGLs and non-HNPGLs/PCCs with a favorable safety profile. Our findings demonstrate that SRS/SBRT achieves high local control rates and should be integrated into the multimodality management of PGL/PCCs, particularly for patients with locally unresectable or advanced diseases. Given its high precision and the convenience of a condensed fractionation schedule, SRS/SBRT represents a safe and effective treatment strategy for non-HNPGLs. By providing radiographic, symptomatic, and biochemical outcomes, this study adds significant evidence to the limited literature supporting radiation therapy as a standard of care for these rare neuroendocrine tumors.

## Figures and Tables

**Table 1:** Patient-Level Characteristics.

		Non-HNPGLs (*n* = 17)	HNPGLs (*n* = 57)
Age at Diagnosis, median [iqr]		45.1 [17.8, 76.3]	54.0 [21.6, 82.0]
Sex, *n* (%)	Female	4 (23.5)	41 (71.9)
	Male	13 (76.5)	16 (28.1)
Tumor categorization, *n* (%)	Paraganglioma	12 (70.6)	57 (100.0)
	Pheochromocytoma	5 (29.4)	0 (0.0)
Malignancy at initial diagnosis, *n* (%)	Metastatic	5 (29.4)	0 (0.0)
	Non-metastatic	12 (70.6)	57 (100.0)
Family history of PGL/PCC, *n* (%)	No	14 (82.4)	55 (96.5)
	Yes	3 (17.6)	2 (3.5)
Mutation Status, *n* (%)	SDHB	6 (35.3)	1 (1.8)
	SDHC	1 (5.9)	2 (3.5)
	SDHD	0 (0.0)	1 (1.8)
	None	3 (17.6)	4 (7.0)
	Not tested/not available	7 (41.2)	49 (86.0)
Prior EBRT *, *n* (%)	No	14 (82.4)	56 (98.2)
	Yes	3 (17.6)	1 (1.8)
Systemic therapy prior to EBRT ^†^, *n* (%)	CVD	5 (29.4)	0 (0.0)
	Capecitabine/Temozolomide	3 (17.6)	0 (0.0)
	Other chemotherapies	1 (5.9)	1 (1.8)
	^131^I-MIBG	2 (11.8)	0 (0.0)
	PRRT	2 (11.8)	0 (0.0)
	Targeted therapies	1 (5.9)	0 (0.0)
Systemic therapy following EBRT ^†^, *n* (%)	CVD	1 (5.9)	0 (0.0)
	Capecitabine/Temozolomide	2 (11.8)	0 (0.0)
	Other chemotherapies	0 (0.0)	1 (1.8)
	^131^I-MIBG	2 (11.8)	0 (0.0)
	PRRT	1 (5.9)	0 (0.0)
	Targeted therapies	3 (17.6)	0 (0.0)

Abbreviations: Non-HNPGL, non-head and neck paraganglioma; HNPGL, head and neck paraganglioma; EBRT, external beam radiation therapy; iqr, interquartile range; CVD, cyclophosphamide/vincristine/dacarbazine regimen; ^131^I-MIBG, ^131^I-metaiodobenzylguanidine therapy; PRRT, Peptide Receptor Radionuclide Therapy; * patients received EBRT to PGL/PCC at outside institutions; † prior to and following first EBRT course for each patient. Patients could have received more than one therapy.

**Table 2:** Lesion-level characteristics and treatment details.

		Non-HNPGLs (*n* = 67)	HNPGLs (*n* = 62)
Sites treated, site, *n*	Primary Lesion	Bone, 3 Adrenal gland, 2 Mediastinum, 2 Retroperitoneum, 1	Jugular foramen, 51 Carotid body, 6 Vagal nerve, 1 Tympanic cavity/middle ear, 1 Unspecified neck, 2
	Metastatic Lesion	Bone, 54 Adrenal gland, 1 Retroperitoneum, 1 Lymph Nodes, 2 Lung, 1	Carotid body, 1
Tumor volume	Median, cc (range)	37.2 (0.6–659.0)	8.8 (0.3–226.0)
	Missing, *n* (%)	8 (11.9)	2 (3.2)
Indication for EBRT, *n* (%)	Definitive (no resection)	58 (86.6)	43 (69.4)
	Recurrence following resection	6 (9.0)	15 (24.2)
	Residual following resection	3 (4.5)	9 (14.5)
SRS/SBRT, *n* (%)	Yes	34 (50.7)	57 (91.9)
	No	33 (49.3)	5 (8.1)
Prescribed SRS/SBRT Regimens, *n* (%)	14–25 Gy in 1 Fx	19 (55.8)	25 (43.9)
	20–28 Gy in 2 Fx	2 (5.9)	5 (8.8)
	18–45 Gy in 3 Fx	8 (23.5)	24 (42.1)
	25–40 Gy in 5 Fx	4 (11.8)	3 (5.3)
	36 Gy in 6 Fx	1 (2.9)	0 (0.0)
Prescribed non-SRS/SBRT Regimens, *n* (%)	30–40 Gy in 10 Fx	15 (45.5)	0
	37.5 Gy in 15 Fx	3 (9.1)	0
	20–25 Gy in 5 Fx	6 (18.2)	0
	8 Gy in 1 Fx	4 (12.1)	0
	Other	5 (15.2)	5 (100.0)
SRS/SBRT Treatment Year, *n* (%)	1998–2005	2 (3.0)	16 (25.8)
	2006–2015	15 (22.4)	28 (45.2)
	2016–2025	17 (25.4)	13 (21.0)
Non-SRS/SBRT Treatment Year, *n* (%)	1998–2005	0 (0.0)	1 (1.6)
	2006–2015	15 (22.4)	1 (1.6)
	2016–2025	18 (26.9)	3 (4.8)
Symptomatic Outcomes, *n* (%)	Improved	14 (20.9)	23 (37.1)
	Stable	25 (37.3)	20 (32.3)
	Worsened	11 (16.4)	2 (3.2)
	NR	14 (20.9)	9 (14.5)
	Asymptomatic	3 (4.5)	8 (12.9)

Abbreviations: Non-HNPGL, non-head and neck paraganglioma; HNPGL, head and neck paraganglioma; EBRT, external beam radiation therapy; SRS/SBRT, stereotactic radiosurgery/stereotactic body radiation therapy; Fx, fraction; NR, not reported.

**Table 3:** Biochemical response among five patients with elevated baseline labs.

Tumor Category	Biochemical Test	Pre-EBRT Measurement	Post-EBRT Measurement
Non-HNPGL	CgA (nl: <93 ng/mL)	506	212
Non-HNPGL	CgA (nl: <93 ng/mL)	786	2756
Non-HNPGL	CgA (nl: <225 ng/mL)	3980	3380
	PMN (nl: <0.50 nmol/L)	36	34
PCC	CgA (nl: <93 ng/mL)	2338	1385
PCC	CgA (nl: <93 ng/mL)	194,600	31,140
	PMN (nl: <0.50 nmol/L)	0.61	0.46

Abbreviations: Non-HNPGL, non-head and neck paraganglioma; PCC, pheochromocytoma; EBRT, external beam radiation therapy; CgA, chromogranin A; PMN, plasma free metanephrine; nl, normal.

**Table 4:** Treatment outcomes of non-HNPGLs and HNPGLs.

	Non-HNPGLs	HNPGLs
	*n* = 59 lesions	*n* = 62 lesions
Local Control, % (95% CI)		
2-year	100	100
5-year	95.3 (85.7–99.2)	100
	*n* = 17 patients	*n* = 62 patients
Distant Progression-Free Survival, % (95% CI)		
1-year	86.9 (71.4–100)	96.3 (91.5–100)
2-year	63.7 (42.5–95.5)	96.3 (91.5–100)
5-year	46.5 (25.5–84.7)	96.3 (91.5–100)
Median Time to Progression, months (95% CI)	37.6 (23.8–NA)	NR
Overall Survival, % (95% CI)		
1-year	92.3 (78.9–100)	98.2 (94.6–100)
2-year	83.9 (65.8–100)	98.2 (94.6–100)
5-year	52.5 (29.1–94.6)	98.2 (94.6–100)
Median Overall Survival, months (95% CI)	131 (40.2–NA)	NR

Note: eight non-HNPGL lesions were excluded in local control outcome analysis due to no follow-up imaging available. Abbreviations: Non-HNPGL, non-head and neck paraganglioma; HNPGL, head and neck paraganglioma; NR, not reached; CI, confidence interval.

**Table 5:** Case reports of EBRT to non-HNPGLs and PCCs.

Author, Year	Sex/Age (Years)	Lesions, *n* (Type)	Site(s) of EBRT	Dose/Fraction	Outcomes
Drasin, 1978 [16]	M/70	2 (PCC)	vertebral metastases	37 Gy/22 36 Gy/18	Local failure at both sites; slowed distant progression; significant pain relief
Siddiqui, 1988 [17]	M/29	3 (PCC)	abdomen primary, femur metastases, vertebral metastases	31 Gy/18 13 Gy/1 25 Gy/5	LC at abdomen maintained at 12 months; distant progression after EBRT; resolution of neurologic symptoms
Olson, 1989 [18]	F/73	2 (PCC)	vertebral metastases, seventh rib metastases	30 Gy 30 Gy	Local failure at vertebral metastases; LC at rib maintained at 15 months; continued distant progression after EBRT; resolution of radiculopathy/weakness and chest wall pain lesion
Jindel, 1992 [19]	M/29	2 (PGL)	spinal extradural metastases, frontal lobe skull metastases	30 Gy/10 47 Gy/20	LC at all sites; continued distant progression after EBRT; symptom relief (paraparesis, incontinence, skull swelling)
Mertens, 1993 [20]	M/23	2 (PGL)	vertebral metastases	50 Gy/25 40 Gy/20	LC maintained at 24 months; continued distant progression after EBRT; temporary pain relief
Brodkey, 1995 [21]	M/32	2 (PGL)	vertebral metastases	50 Gy/25 44 Gy/22	Local and distant control maintained at 24 months; resolution of myelopathy
Pickard, 1995 [22]	M/8	1 (PCC)	renal hilar primary	NR	Local and distant control maintained at 48 months; normotensive after EBRT
Teno, 1996 [23]	F/44	2 (PCC)	sacral metastases, thoracic vertebral metastases	60 Gy 20 Gy/5	Hypertension exacerbation after thoracic EBRT resulting in abrupt stop of treatment; continued distant progression; minimal relief of hip pain
Yu, 1996 [24]	F/30	2 (PCC)	liver metastasis, parasellar metastasis	23 Gy 25 Gy/10	LC at liver maintained at 12 months; LC at parasellar lesion maintained at 26 months; resolution of cranial nerve deficits and symptoms
Hamilton, 2000 [25]	M/16	3 (PGL)	sternum bone metastasis, vertebral metastases, vertebral metastases	40 Gy/15 30 Gy/10 41 Gy/13	LC at sternum lesion; continued distant progression; no improvement in paraplegia
	M/17	1 (PGL)	vertebral metastases	20 Gy/5	New onset of symptoms with progression (cord compression, urinary incontinence, sacral paranesthesia, sciatic radiculopathy)
Hruby, 2000 [26]	F/77	1 (PGL)	vertebral metastases	40 Gy/16	Local failure; continued distant progression; no improvement in lower back pain
Taue, 2001 [27]	M/47	1 (PGL)	vertebra metastasis	50 Gy	Local and distant control maintained at 20 months; resolution of right-sided paraplegia; normalization of adrenaline and dopamine levels
U-King-Im 2002 [28]	F/32	3 (PGL)	vertebral metastases	30 Gy in 10	No distant progression over 3 years after EBRT; relief of thoracic pain
Tekautz, 2003 [29]	F/8.3	1 (PGL)	retroperitoneal primary	36 Gy in 4	Local and distant control maintained at 10.7 years
	M/15.7	1 (PGL)	retroperitoneal primary	50.8 Gy	Local and distant control maintained at 4.6 years
Lazaro, 2003 [30]	F/29	1 (PGL)	vertebral metastases	40 Gy	Pain improvement
Edström Elder, 2003 [31]	2 M, 2 F/42.5	8 (PGL)	osseous metastases	Median 20 Gy	Temporary symptom relief across all lesions; reduced catecholamine levels
Yoshida, 2004 [32]	M/24	3 (PGL)	lymph nodes metastases, para-aortic metastases, lymph nodes metastases	60 Gy 50 Gy 50 Gy	LC at pelvic lymph nodes; continued distant progression; decrease in catecholamine levels
Nitsche, 2005 ^†^ [33]	M/55	1 (PGL)	cerebellar bone primary *	45 Gy/25	Local and distant control maintained at 23 months
Wong, 2005 [34]	F/49	1 (PGL)	abdomen primary	50 Gy/28	Local and distant control; pain improvement
Krych, 2006 [35]	NR	1 (PGL)	retroperitoneal PGL	45 Gy	Local failure at 21 months; continued distant progression
Laufer, 2007 [36]	F/69	1 (PGL)	sacral metastases	30 Gy/10	Local and distant control; improvement in back pain
Kasliwal, 2008 [37]	M/47	1 (PGL)	vertebra metastasis *	NR	Local failure at 24 months; distant progression at 30 months
Trombetta, 2008 ^†^ [38]	M/64	3 (PGL)	T9-L1 metastases, suprasellar metastases, T6 vertebra metastasis	39 Gy/13 50.4 Gy/28 37.5 Gy/15	LC at all sites maintained shortly after EBRT; stabilization of headaches
Haresh, 2009^†^ [39]	M/17	2 (PGL)	sellar primary *, bone metastasis	50 Gy/25 NR	Distant progression followed by EBRT to bones; acute symptomatic improvement
Lehmen, 2010 [40]	M/71	2 (PGL)	vertebral metastases	30 Gy/10	Significant improvement in upper extremity weakness and gait with EBRT and surgery
Richter, 2011 [41]	F/16	1 (PGL)	L1 vertebra metastasis	50 Gy	Local and distant control maintained at 10 years; resolution of symptoms
Garibaldi, 2011 ^†^ [42]	M/55	2 (PGL)	left pontocerebellar angle, mediastinal paraaortic	50.40 Gy/28 55.80 Gy/31	Local and distant control maintained at 14 months; normalization of urinary catecholamine levels
Simpson, 2012 [43]	F/49	1 (PGL)	vertebral primary	45 Gy	Local and distant control maintained at 2 years; resolution of symptoms (HTN, palpitations, sweating, arm pain); normetanephrine levels decreased
Sasaki, 2013 [44]	M/72	2 (PGL)	cervical vertebral metastases *, lumbar vertebral metastases	NR	LC at both sites maintained at 3 months; improvement of pain and weakness in upper limbs with CK and surgery
Li, 2014 [45]	M/47	2 (PGL)	liver metastasis, pelvic metastasis	50.4 Gy 50.4 Gy	Local failure after EBRT; persistent local progression in both irradiated sites
Choi, 2015 [46]	F/47	1 (PCC)	abdomen metastasis	50.4 Gy	Local and distant control maintained after 11.2 years
Heijneman, 2015 [47]	M/36	2 (PGL)	retroperitoneal primary, vertebral metastases	NR 8 Gy	Continued distant progression; fatal catecholamine-induced cardiomyopathy
Wu, 2015 [48]	M/44	1 (PGL)	S1-S3 vertebrae with L4–L5 canal extension primary	50 Gy/25	Alive with no evidence of disease progression at 1-year follow up; definite recovery of motion, sensation, and autonomic function
Miyahara, 2017 [49]	M/57	1 (PCC)	left parietal lobe metastases *	24 Gy/1	LC maintained at 1 year; symptoms of aphasia and hemiplegia worsened after 1 year; radiation necrosis after 1 year
Kapetanakis, 2017 [50]	M/48	1 (PGL)	vertebral metastases *	NR	Distant progression in lumbar spine; biochemical improvement during 6-month follow-up; significant improvement in pain
Jia, 2018 [51]	1 F, 3 M/46.5	4 (PGL)	osseous metastases	NR	Local and distant control maintained at median 40 months
	4 M/35.5	4 (PGL)	osseous metastases	NR	Local failure at median 24 months
	M/47	1 (PGL)	osseous metastases	NR	Distant progression at 48 months. Alive with disease
Fadiga, 2019 [52]	F/66	1 (PCC)	T10 vertebra metastasis	30 Gy/10	LC maintained at 5 years; elevated chromogranin A and urinary metanephrine after 4 months
Jha, 2019 [53]	NR	1 (PCC)	vertebral metastases	30 Gy/10	Widespread distant progression
	NR	1 (PGL)	vertebral metastases	54 Gy/30	Local failure at 14 months
	NR	2 (PGL)	osseous metastases	32.5 Gy/13 NR	Widespread distant progression
Elenkova, 2019 [54]	M/23	1 (PGL)	mediastinal primary	55.8 Gy	Local failure 5 years after EBRT
Alzahrani, 2020 [55]	M/24	1 (PGL)	lumbar spine metastases	NR	Alive with stable and slow progression of metastases at 14 years
Grambozov, 2020 [56]	F/30	2 (PGL)	vertebral metastases	50 Gy/2.5 40 Gy/2	LC maintained at 1 year
Grant, 2020 [57]	M/52	2 (PCC)	vertebral metastases *	24 Gy/1 16 Gy	Widespread progression of metastases after 2 years
Stojanoski, 2021 ^†^ [58]	M/31	1 (PGL)	bilateral sellar/parasellar primary *	12 Gy/1	Local failure at 48 months; received salvage SRS; distant progression 3 months after 2nd SRS
Kitagawa, 2021 [59]	M/54	6 (PGL)	vertebral metastases	40 Gy/20	LC but progressive systemic disease
Gonzalez, 2021 [60]	NR/27	1 (PCC)	right adrenal gland primary *	24 Gy/3	Symptomatic control of headache and blood pressure stabilization
Zouraq, 2022 [61]	M/37	1 (PGL)	bilateral internal iliac spaces primary	60 Gy/30	LC maintained at 6 months; high level of urinary catecholamines maintained; improvement of hypertension
Ghaisas, 2022 ^†^ [62]	M/20	1 (PGL)	sellar-suprasellar region primary	40 Gy/20	LC maintained at 3 years; improvement in visual acuity
Carafone, 2023 [63]	M/64	1 (PGL)	right atrial wall/atrioventricular groove primary	45 Gy/25	Significant decrease in catecholamine level following EBRT
Tabb, 2023 [64]	F/40	2 (PGL)	osseous metastases	30 Gy/10 8 Gy/1	Complete neurological recovery, physical function, and pain relief at 13 months
Du, 2023 [65]	M/57	1 (PCC)	T5 vertebra metastasis	35 to 45 Gy	Deceased after 5 months
	2 M/44	2 (PCC)	vertebral metastases	35 to 45 Gy	LC maintained after median 55.5 months. Alive with disease
	2 M/ 53.5	2 (PGL)	vertebral metastases	35 to 45 Gy	LC maintained after median 49.5 months. Alive with disease
Pontoriero, 2024 [66]	NR/60	1 (PGL)	cervical spine primary *	20 Gy/5	Progression-free survival 96 months; overall survival 156 months
Yu, 2024 [67]	M/71	1 (PGL)	left femur metastases	30 Gy/10	Reduction in normetanephrine level after 3 months; intermittent pain with weight bearing remained
Bajagain, 2024 ^†^ [68]	M/52	1 (PGL)	right cavernous sinus primary *	15 Gy/1	LC maintained at 2-year follow up, no progression
Treffy, 2025 [69]	M/55	2 (PGL)	vertebral metastases	NR 50 Gy/25	LC maintained at 11 months; complete recovery of motor strength after 11 months

Note: patients and/or lesions were aggregated across each case report if the tumor type, EBRT site, and outcomes were the same. If aggregated, median age is reported; Abbreviations: Non-HNPGL, non-head and neck paraganglioma; PGL, paraganglioma; PCC, pheochromocytoma; EBRT, external beam radiation therapy; SRS/SBRT, stereotactic radiosurgery/stereotactic body radiation therapy; LC, local control; NR, not reported; HTN, hypertension; CK, CyberKnife. * Lesions treated with SRS/SBRT; † sellar, cerebellar, and cavernous sinus PGLs were categorized as non-HNPGLs because they were ectopic intracranial paragangliomas.

**Table 6:** Cohort studies of EBRT to non-HNPGLs and PCCs.

Author, Year	Median Age, Years (Range)	Patient/Lesions, *n*	Site(s) of EBRT	Dose/Fraction	Outcomes
Massey, 1992 [13]	36.5	4/11	Bone, 10 Soft tissue, 1	Median 33 Gy/11	90% LC; 89% symptomatic control; 4/4 died from widespread distant progression; 1-year, 5-year OS: 100%, 25%; Vertebral collapse in one patient.
Fishbein, 2012 [7]	NR	17/22	Bone, 11 Abdominal, 5 Lung and thorax, 4 Other, 2	Median 40 Gy/17	1-year LC: 76%; 76% symptomatic control; TTP: 11.9 months; 3/3 catecholamine ↓; Overall mild nausea and diarrhea. 3 patients with SDHB mutation.
Vogel, 2014 [8]	Mean 39.5 (12–59)	24/47	Bone, 40 Abdominal, 3 CNS, 4	Mean 31.8 Gy/3.3	87% LC; SRS LC: 100% (*n* = 4); 81% symptomatic control; 75% distant progression; 4/6 CgA ↓, 3/6 PMN ↓, 3/4 UMN ↓; 87.5% alive at median 52 months; two upper gastrointestinal acute G3 events. 9 patients with SDHB mutation.
Breen, 2018 [9]	33 (11–80)	41/107	Bone, 74 Soft tissue, 32 Liver, 1	Median BED_3_ = 75.7 Gy Median BED_10_ = 53 Gy	5-year LC: 81%, SRS LC: 100% at median 3 year (*n* = 11); 94% symptomatic control; 5-year dPFS: 30%; 5/7 metanephrine ↓, 5-year OS: 65%, two late G3 events. 12 patients with SDHB mutation.
Mesko, 2019 [14]	58 (46–68)	7 /16	Cervical spine, 3 Thoracic spine, 6 Lumbar spine, 5 Sacrum, 2	Median 27 Gy/3	SRS LC 93.7%; 1-year, 2-year OS: 71.4%, 42.9%
Gu, 2023 [10]	44.9 (15–67)	14/17	Bone, 3 Brain, 1 Lung, 3 Abdominal, 10	Median BED_3_ = 130 Gy Median BED_10_ = 74.4 Gy	94% LC; 91% symptomatic control; TTP: 5.2 months, 28.5% distant progression; 2-year OS: 78%. 4 patients with SDHB mutation.
Terakawa, 2023 [11]	55 (52–72)	3/4	Lymph nodes, 1 Vertebral spine, 2 Retroperitoneum, 1	Median BED_10_ = 46 Gy	2-year LC: 100%; 100% normetanephrine ↓

Abbreviations: Non-HNPGL, non-head and neck paraganglioma; PCC, pheochromocytoma; EBRT, external beam radiation therapy; SRS, stereotactic radiosurgery; LC, local control; OS, overall survival; dPFS, distant progression-free survival; CgA, chromogranin A; PMN, plasma free metanephrine; UMN, urinary fractionated metanephrine; NR, not reported; TTP, time to distant progression; BED_3_, biologically effective dose (α/β = 3); BED_10_, biologically effective dose (α/β = 10); ↓, decreased levels.

## Data Availability

The dataset is available upon request from the authors.

## Abbreviations

The following abbreviations are used in this manuscript:

PGL	Paraganglioma
PCC	Pheochromocytoma
HNPGL	Head and neck paraganglioma
Non-HNPGL	Non-head and neck paraganglioma
EBRT	External Beam Radiation Therapy
SRS	Stereotactic Radiosurgery
SBRT	Stereotactic Body Radiation Therapy
BED_10/3_	Median biologically effective dose (α/β = 10 or 3)
UMN	Urinary fractionated metanephrine
PMN	Plasma free metanephrine
CgA	Chromogranin A
CTCAE	Common Terminology Criteria for Adverse Events
IQR	Interquartile range
LC	Local control
dPFS	Distant progression-free survival
OS	Overall survival
CI	Confidence interval
CVD	Cyclophosphamide/vincristine/dacarbazine regimen
^131^I-MIBG	^131^I-metaiodobenzylguanidine therapy
PRRT	Peptide receptor radionuclide therapy
Fx	Fraction
NR	Not reported or not reached
nl	Normal
TTP	Time to distant progression
HTN	Hypertension
CK	CyberKnife

## Appendix A. PubMed/MEDLINE Search Strategy

(“phaeochromocytoma*”[tiab] OR “pheochromocytoma”[MeSH Terms] OR “pheochromocytoma*”[tiab] OR “paraganglioma”[MeSH Terms] OR “paraganglioma*”[tiab])

AND

(“proton therapy”[MeSH Terms] OR “proton beam therapy”[tiab] OR “proton therapy”[tiab] OR “external beam”[tiab] OR “Radiotherapy”[MeSH Terms] OR “3D conformal”[tiab] OR “Radiotherapy, Conformal”[mesh] OR “conformal radi*”[tiab] OR “stereotactic radiosurgery”[tiab] OR “Radiotherapy, Intensity-Modulated”[Mesh] OR “intensity modulated”[tiab] OR “Radiosurgery”[Mesh] OR “stereotactic radiotherapy”[tiab:~3] OR “stereotactic radiation”[tiab:~3] OR LINAC[tiab] OR “gamma knife”[tiab] OR gammaknife[tiab] OR cyberknife[tiab] OR “cyber knife”[tiab] OR “linear accelerator radi*”)
